# Efficacy and Safety of Rifaximin Versus Placebo or Other Active Drugs in Critical ill Patients With Hepatic Encephalopathy

**DOI:** 10.3389/fphar.2021.696065

**Published:** 2021-10-08

**Authors:** Xianghui Han, Zhanyang Luo, Wenyi Wang, Peiyong Zheng, Tian Li, Zubing Mei, Jianyi Wang

**Affiliations:** ^1^ Department of Liver Disease, Shuguang Hospital Affiliated to Shanghai University of Traditional Chinese Medicine, Shanghai, China; ^2^ Institute of Chinese Traditional Surgery, Longhua Hospital Affiliated to Shanghai University of Traditional Chinese Medicine, Shanghai, China; ^3^ Institute of Digestive Diseases, Longhua Hospital Affiliated to Shanghai University of Traditional Chinese Medicine, Shanghai, China; ^4^ School of Basic Medicine, Fourth Military Medical University, Xi’an, China; ^5^ Department of Anorectal Surgery, Shuguang Hospital Affiliated to Shanghai University of Traditional Chinese Medicine, Shanghai, China; ^6^ Anorectal Disease Institute of Shuguang Hospital, Shanghai, China

**Keywords:** rifaximin, hepatic encephalopathy, efficacy, safety, meta-analysis

## Abstract

**Objective:** Rifaximin has been approved for use as a first-line therapy for secondary prophylaxis of hepatic encephalopathy (HE). This article is to update existing evidence on efficacy and safety of rifaximin treatment and prevention for HE.

**Methods:** We systematically searched multiple databases until January 31 2021. The studies compared rifaximin vs. placebo or other active drugs (i.e., nonabsorbable disaccharides, other antibiotics, L-ornithine-L-aspartate (LOLA), and probiotics) for patients with overt HE (OHE), minimal HE (MHE), and recurrent HE.

**Results:** Twenty-eight randomized controlled trials with a total of 2979 patients were included. Compared with the controls, rifaximin significantly reduced HE grade (OHE: RR = 1.11, 95% CI = 1.02–1.21), improved the cognitive impairments (MHE: RR = 1.82, 95% CI = 1.12–2.93) and prevented the risk of HE recurrent episodes (RR = 1.33, 95% CI = 1.18–1.49). No statistical difference was observed in mortality between rifaximin and their controls (RR = 0.82, 95% CI = 0.54–1.24). The incidence of total adverse events in rifaximin-treated groups was significantly lower than that in the controls during the treatment period (RR = 0.73, 95% CI = 0.54–0.98). In addition, rifaximin treatment was better than other active drugs in improving psychometric indicators (mental state, flapping tremor and portosystemic encephalopathy (PSE) index) and reducing the risk of rehospitalization in HE patients.

**Conclusion:** Rifaximin therapy is effective and well-tolerated in different types of HE, which might be recommended as an alternative to conventional oral drugs in clinical settings.

## Highlights

What is the current knowledge on the topic?
   Rifaximin has been approved for use as a first-line therapy for secondary prophylaxis of hepatic encephalopathy (HE).
What question did this study address?
   This article is to update existing evidence on efficacy and safety of rifaximin treatment and prevention for HE.
What does this study add to our knowledge?
   Compared with the controls, rifaximin significantly reduced HE grade, improved the cognitive impairments, and prevented the risk of HE recurrent episodes. No statistical difference was observed in mortality between rifaximin and their controls. The incidence of total adverse events in rifaximin-treated groups was significantly lower than that in the controls during the treatment period. In addition, rifaximin treatment was better than other active drugs in improving psychometric indicators (mental state, flapping tremor and PSE index) and reducing the risk of rehospitalization in HE patients.
How might this change clinical pharmacology or translational science?
   Rifaximin therapy is effective and well-tolerated in different types of HE, which might be recommended as an alternative to conventional oral drugs in clinical settings.

## Introduction

Hepatic encephalopathy (HE) is a frequent and serious complication of end-stage liver cirrhosis due to liver insufficiency or portosystemic shunting (American Association for the Study of Liver, 2014). According to the severity of manifestations, HE is subdivided into minimal and overt (grade I–IV) types by the West Haven Criteria (Ferenci, 2017). Overt HE (OHE) presents abnormal blood ammonia levels and neurological symptoms including asterixis, deterioration of mental state, and even coma, which leads to a burden on health care systems and a notable decline in quality of life (Weissenborn et al., 2005). Minimal HE (MHE) mainly encompasses cognitive impairment, such as attention, alertness, orientation, and learning processes, which is detected through changes in neuro-psychometric (NP) or critical flicker frequency (CFF) tests. It is reported that OHE occur in 30–40% of patients with liver cirrhosis and MHE occurred in 20–80% of those with cirrhosis during their clinical course (Romero-Gómez et al., 2007). When the first episode of OHE is not actively treated, about 18.1% of patients re-entered hospitals within 30 days. HE recurrence can aggravate clinical symptoms and increase rehospitalization and mortality (Tapper et al., 2016; Wang et al., 2021).

Currently, treatment options for OHE and MHE include nonabsorbable disaccharides, such as lactulose and lactitol; antibiotics that act in intestinal lumen, such as rifaximin, paromomycin, and neomycin; and drugs that favour extrahepatic metabolism of ammonium, such as l-ornithine-l-aspartate (LOLA) and probiotics (Ridola et al., 2018). Rifaximin, which is also called xifaxan, is derived from rifamycin SV, and its chemical name is 4-deoxy-4ʹ-methylpyrido-(1ʹ,2ʹ-1,2)-imidazo-(5,4C)-rifamycin SV (Scarpignato and Pelosini, 2005). As an oral drug with poor absorption and broad-spectrum antimicrobial activity, rifaximin was approved by the US Food and Drug Administration for use in HE treatment (Al Sibae and McGuire, 2009). In clinics, rifaximin has been recommended as the first choice for prevention of HE recurrence or used as an add-on to nonabsorbable disaccharides to treat the patients with OHE or MHE (Bajaj et al., 2011).

To date, 10 meta-analyses including seven systematic reviews (Jiang et al., 2008; Eltawil et al., 2012; Hu and Tang, 2013; Wu et al., 2013; Kimer et al., 2014; Wang, 2015; Zhuo et al., 2019) and three network meta-analyses (Zhu et al., 2015; Cai et al., 2018; Dhiman et al., 2020), have been published on efficacy or safety of rifaximin for HE treatment. Most of these meta-analyses focused on one control arm or one type of HE, and their results seem equivocal and inconsistent. Therefore, we searched all eligible randomized controlled trials (RCTs) on rifaximin treatment for patients with HE from 1991 to 2020, and performed a meta-analysis to evaluate comprehensively the efficacy and safety of rifaximin vs. placebo or other active drugs (nonabsorbable disaccharides, other antibiotics, LOLA and probiotics) for pharmacological management of overt and minimal HE or prevention of recurrent HE. This meta-analysis updated existing evidence on the extensive clinical use of rifaximin for treatment of different types of HE.

## Materials and Methods

This meta-analysis was conducted in accordance with the Preferred Reporting Items for Systematic Reviews and Meta-Analyses (Moher et al., 2009; Zhang et al., 2021a; Zhang et al., 2021b) and registered in the PROSPERO database (registration number: CRD42020206066).

### Search Strategy

The methodology is performed as previously described (Li et al., 2021). We systematically searched PubMed, embase, Web of Science, Cochrane Library, and several Chinese databases (CNKI, VIP, and Wanfang databases) until January 10, 2021. We used the following MeSH terms: (“Rifaximin*” OR “4-Deoxy-4'-methylpyrido (1',2'-1,2) imidazo (5,4C) rifamycin*” OR “L 105*” OR “L105*” OR “L-105*” OR “redactiv*” OR “xifaxan*” OR “normix*” OR “rifamycin*”) AND (“Hepatic Encephalopathy*” OR “Hepatic Stupor*” OR “Hepatic Coma*” OR “Portal systemic encephalopathy*” OR “Encephalopathy, Hepatic*” OR “Encephalopathy, Portal-Systemic*,” *etc*.). The full search strategy is provided in Supplementary Table S1. Two reviewers independently assessed the eligibility of the titles and abstracts in all articles.

### Inclusion and Exclusion Criteria

According to PICOS criteria, two reviewers (ZL and ZM) independently selected and evaluated the studies through different databases. The studies were included if they met the following criteria: (American Association for the Study of Liver, 2014): Participant: adults with an age of at least 18 years; (Ferenci, 2017); Intervention: assessment of the efficacy and safety of rifaximin on patients with liver cirrhosis and overt, minimal, or recurrent HE; (Weissenborn et al., 2005); Comparator: rifaximin compared with other interventions, such as placebo and other active drugs (nonabsorbable disaccharides, other antibiotics, LOLA, or probiotics); (Romero-Gómez et al., 2007); Outcomes: primary outcomes, including OHE improvement, MHE reversal, prevention of recurrent HE, mortality, and adverse effects; secondary outcomes, including blood ammonia level, mental state, flapping tremor, rehospitalization, and portosystemic encephalopathy (PSE) index; (Tapper et al., 2016); Study design: RCTs with either multicentre or single-centre design. Exclusion criteria were: (American Association for the Study of Liver, 2014): trials conducted among children patients; (Ferenci, 2017); non-controlled clinical trials and phase I clinical trials; (Weissenborn et al., 2005); trials that assessed the efficacy and safety of rifaximin combined with other active drugs; (Romero-Gómez et al., 2007); trials including patients with psychiatric illness, with undercurrent infections, with hypersensitivity to rifaximin or intolerance to other active drugs.

Following the Cochrane Handbook guidelines, data were independently extracted from the eligible RCTs by two investigators (ZL and ZM). The extracted data included the following information: first author, publication year, study design, type of HE, study groups, treatment duration, number of patients, and outcomes. When disagreement arose, all reviewers discussed the merits of the studies until a consensus was achieved.

### Risk of Bias and Quality Assessment

Risk of bias (ROB) in the eligible RCTs was assessed using ROB version 1.0 in the Cochrane Handbook, including the following domains: random sequence generation, allocation concealment, blinding, incomplete outcome data, and selective reporting. Two reviewers (JW and XH) independently assessed risk of bias in each included trial. We resolved disagreements by consulting a third review author (ZM). Each domain was judged as high, low, or unclear risk of bias.

The quality of evidence for primary and secondary outcomes was assessed using the GRADE (Grading of Recommendations, Assessment, Development and Evaluations) system to arrive at credible conclusions for the reviewers. Results from the included RCTs were potentially downgraded because of risk of bias, indirectness of evidence, inconsistency in results, publication bias, or imprecision of results (Guyatt et al., 2008).

### Definition of Outcomes

OHE improvement, MHE reversal and prevention of recurrent HE, were calculated by the proportion that rifaximin significantly improved HE grade, reversed cognitive impairments, and reduced recurrent HE episodes, respectively. Mortality was analysed by the number of deaths during the treatment period. Adverse effects of rifaximin and other oral therapies assessed in this study were: total adverse events, abdominal pain, diarrhea, nausea, fatigue, and vomiting. Blood ammonia level was detected at the end of the treatment. Rehospitalization was defined as the number of rehospitalization patients due to a disorder or an episode of HE occurrence (Bass et al., 2010). Mental state was scored according to Conn’s classification (Conn et al., 1977). Severity of flapping tremor was graded according to a simplified grading system (Grade 0: no flapping motion; Grade 1: infrequent flapping motion; Grade 2: continuous flapping motion; Grade 3: unable to test) (Paik et al., 2005).

### Statistical Analysis

Statistical analysis was performed using Stata software version 12.0. For discontinuous variables (i.e., OHE improvement, MHE reversal, prevention of recurrent HE, mortality, adverse effects, and rehospitalization), we used RR to assess differences between rifaximin and control interventions. For continuous variables, such as mental state, flapping tremor, and PSE index, we used the mean difference (MD) statistic. As for blood ammonia level, we first converted the data to the same unit (expressed as mg/dL) and analyzed using MD. We used the I^2^ statistic to assess between study heterogeneity as follows: 0–40%: might not be important; 30–60%: might represent moderate heterogeneity; 50–90%: might represent substantial heterogeneity; 75–100%: considerable heterogeneity (Higgins and Greeni, 2011).

We used the random-effect model to analyse all quantitative data. All results were estimated from each trial with a 95% confidence interval (CI). Subgroup analyses were conducted to compare the clinical efficacy, mortality and total adverse events of rifaximin vs*.* placebo or other active drugs treatment for different types of HE. A *p* value of <0.05 indicated statistical significance. Publication bias was assessed by funnel plots and Egger’s test. Funnel plots for outcomes with at least 10 RCTs were generated using the standard error of log(RR) and RR or log(weighted mean difference (WMD)) and WMD. Asymmetric funnel plots and results of Egger’s test with *p* < 0.05 were considered as publication bias.

## Results

### Identification and Selection

**FIGURE 1:**
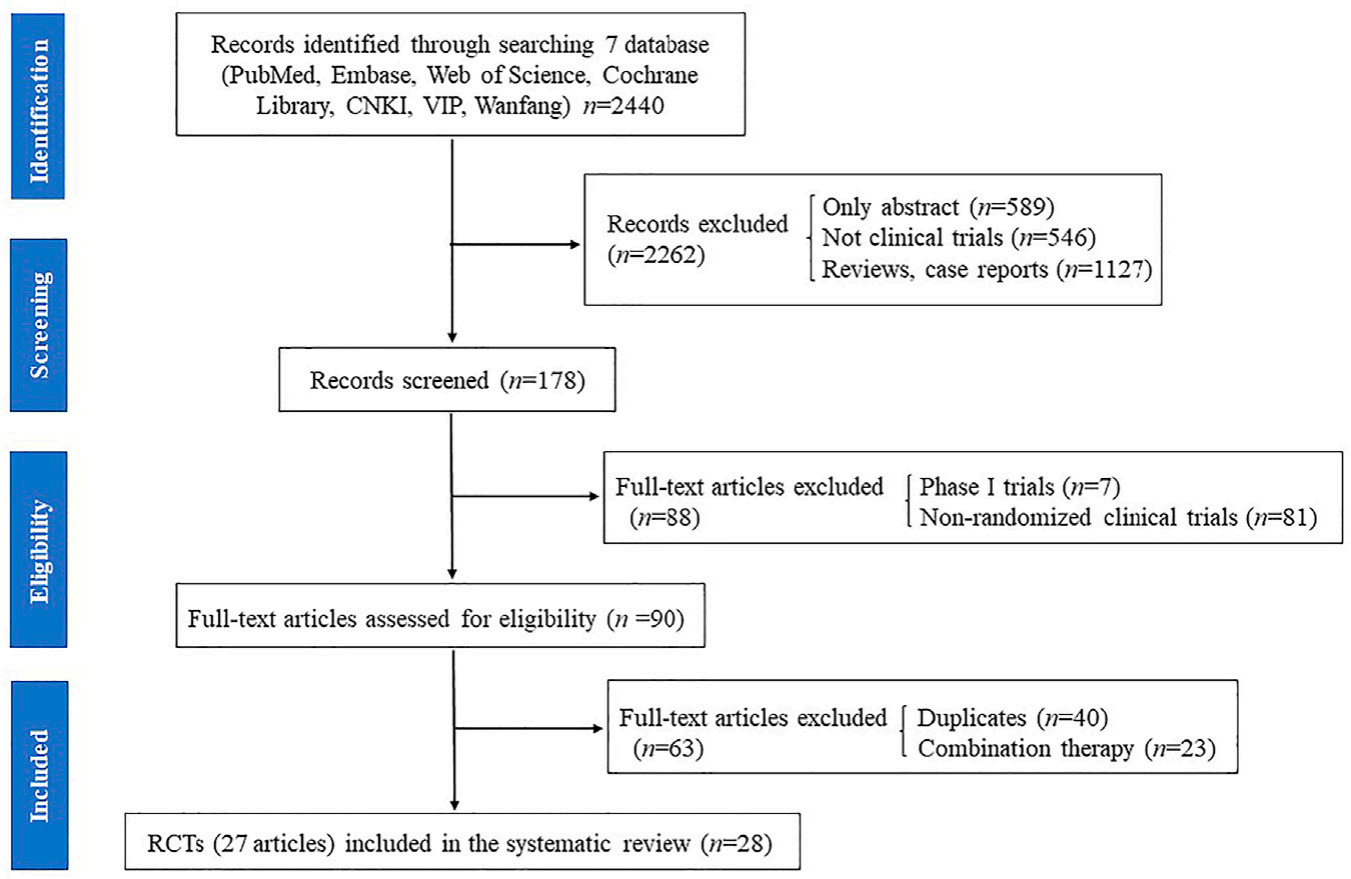
Flowchart of study screen in this meta-analysis.

The results of all records identified in the search are depicted in a flow diagram (Figure 1). A total of 2,440 records were extracted from the electronic literatures, of which 2,262 records were excluded after scanning their titles and abstracts. A total of 178 full-text articles were regarded as potentially eligible for this review. Subsequently, 88 articles, including phase I trials and non-RCTs, were excluded. Afterward, 40 duplicate articles and 23 articles on trials with combined therapy (i.e., rifaximin *plus* lactulose vs. lactulose alone) were excluded. Finally, 27 full-text articles (28 RCTs) were included for assessment in this meta-analysis (Parini et al., 1992; Pedretti et al., 1992; Bucci and Palmieri, 1993; Fera et al., 1993; Festi et al., 1993; Massa et al., 1993; Miglio et al., 1997; Song et al., 2000; Loguercio et al., 2003; Mas et al., 2003; Paik et al., 2005; Riggio et al., 2005; Bass et al., 2010; Bajaj et al., 2011; Sanyal et al., 2011; Sidhu et al., 2011; Neff et al., 2013; Yang, 2013; Sharma et al., 2014; Wahib et al., 2014; Sidhu et al., 2016; Aqeel et al., 2018; Flamm et al., 2018; Higuera-de-la-Tijera et al., 2018; Mekky et al., 2018; Munir et al., 2018; Suzuki et al., 2018).

### Study Characteristics

A description and the characteristics of the 28 RCTs included in this meta-analysis are summarized in Supplementary Table S2. Among these RCTs, 14 conducted double-blind RCTs, and the other 14 were open or one-blind RCTs. Five studies were multicentre trials, and the remainders were single-centre trials. Seven of 28 trials reported the methods of sample size calculation (Mas et al., 2003; Riggio et al., 2005; Bajaj et al., 2011; Sidhu et al., 2011; Sidhu et al., 2016; Higuera-de-la-Tijera et al., 2018; Suzuki et al., 2018). Ten trials assessed the effects of rifaximin on OHE improvement, four and nine trials were related to the effects of rifaximin treatment on MHE reversal and prevention of recurrent HE, respectively. The intervention groups (*n* = 1,403) received rifaximin treatment, whereas the control groups (*n* = 1,576) received placebo (10 trials, *n* = 802) (Riggio et al., 2005; Bass et al., 2010; Bajaj et al., 2011; Sanyal et al., 2011; Sidhu et al., 2011; Neff et al., 2013; Sharma et al., 2014; Aqeel et al., 2018; Flamm et al., 2018; Higuera-de-la-Tijera et al., 2018), or other active drugs such as nonabsorbable disaccharides (15 trials, *n* = 561) (Bucci and Palmieri, 1993; Fera et al., 1993; Festi et al., 1993; Massa et al., 1993; Song et al., 2000; Loguercio et al., 2003; Mas et al., 2003; Paik et al., 2005; Riggio et al., 2005; Yang, 2013; Wahib et al., 2014; Sidhu et al., 2016; Higuera-de-la-Tijera et al., 2018; Munir et al., 2018; Suzuki et al., 2018), other antibiotics (5 trials, *n* = 129) [39,50–53], LOLA (2 trials, *n* = 53) (Sharma et al., 2014; Higuera-de-la-Tijera et al., 2018), or probiotics (1 trial, *n* = 31) (Sharma et al., 2014). The therapeutic dose of rifaximin was 1,100 or 1,200 mg/day, and the intervention duration ranged from 5 days to 1 year. The primary outcomes in these RCTs included OHE improvement (10 RCTs), MHE reversal (4 RCTs), prevention of recurrent HE (9 RCTs), mortality (13 RCTs), and adverse events (17 RCTs). The secondary outcomes included blood ammonia level (12 RCTs), mental state (8 RCTs), flapping tremor (6 RCTs), rehospitalization (3 RCTs), and PSE index (5 RCTs).

### Risk of Bias and Quality Assessment

**FIGURE 2:**
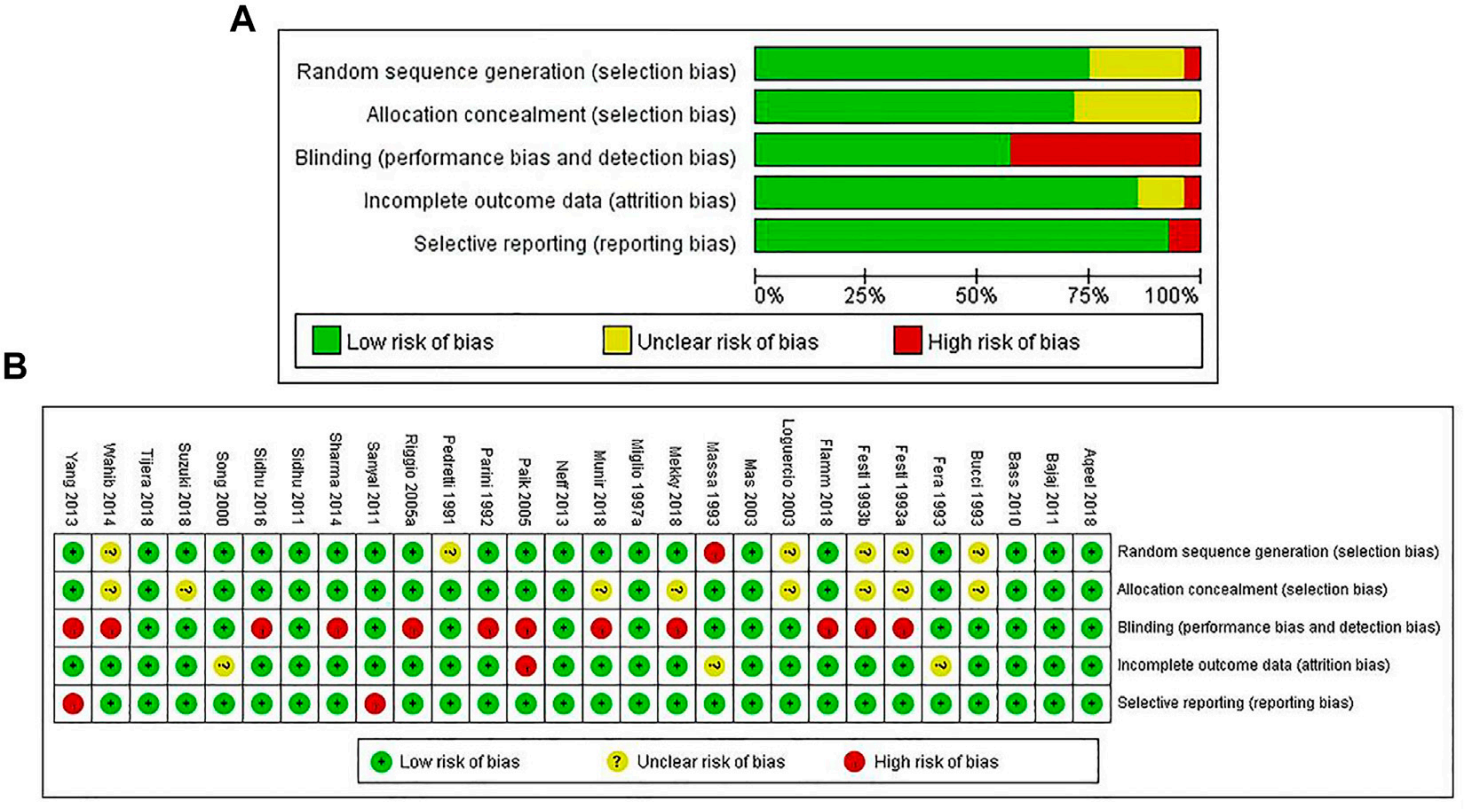
Risk of bias graph: reviewers’ judgments about each risk of bias item presented as percentages across all included studies **(A)**. Risk of bias summary: reviewers’ judgments about each risk of bias item for each included study according to the Cochrane Collaboration’s “Risk of Bias” tool, the green circle with “plus” sign representing low risk of bias, the yellow circle with “question mark” sign representing unclear risk of bias and the red circle with “minus” sign representing high risk of bias **(B)**.

The assessment results of risk of bias showed that 75.0% of the included studies reported adequate random sequence generation, 71.4% reported allocation concealment, 57.1% used blinding, 85.7% avoided incomplete outcome data, and 92.8% avoided selective reporting bias (Figures 2A,B).

The outcomes of clinical efficacy, including OHE improvement, MHE reversal, prevention of recurrent HE, blood ammonia level, mental state, rehospitalization, and PSE index were judged as moderate-quality evidence with heterogeneity I^2^ ranging from 0 to 93.3%. Mortality was judged as moderate-quality evidence (heterogeneity I^2^ = 0.0%). Adverse effects such as abdominal pain, diarrhea, nausea, vomiting, and fatigue were judged as high-quality with heterogeneity I^2^ ranging from 0 to 1%, whereas the outcome of total adverse events was judged as moderate-quality (Supplementary Table S3).

### Publication Bias

**FIGURE 3:**
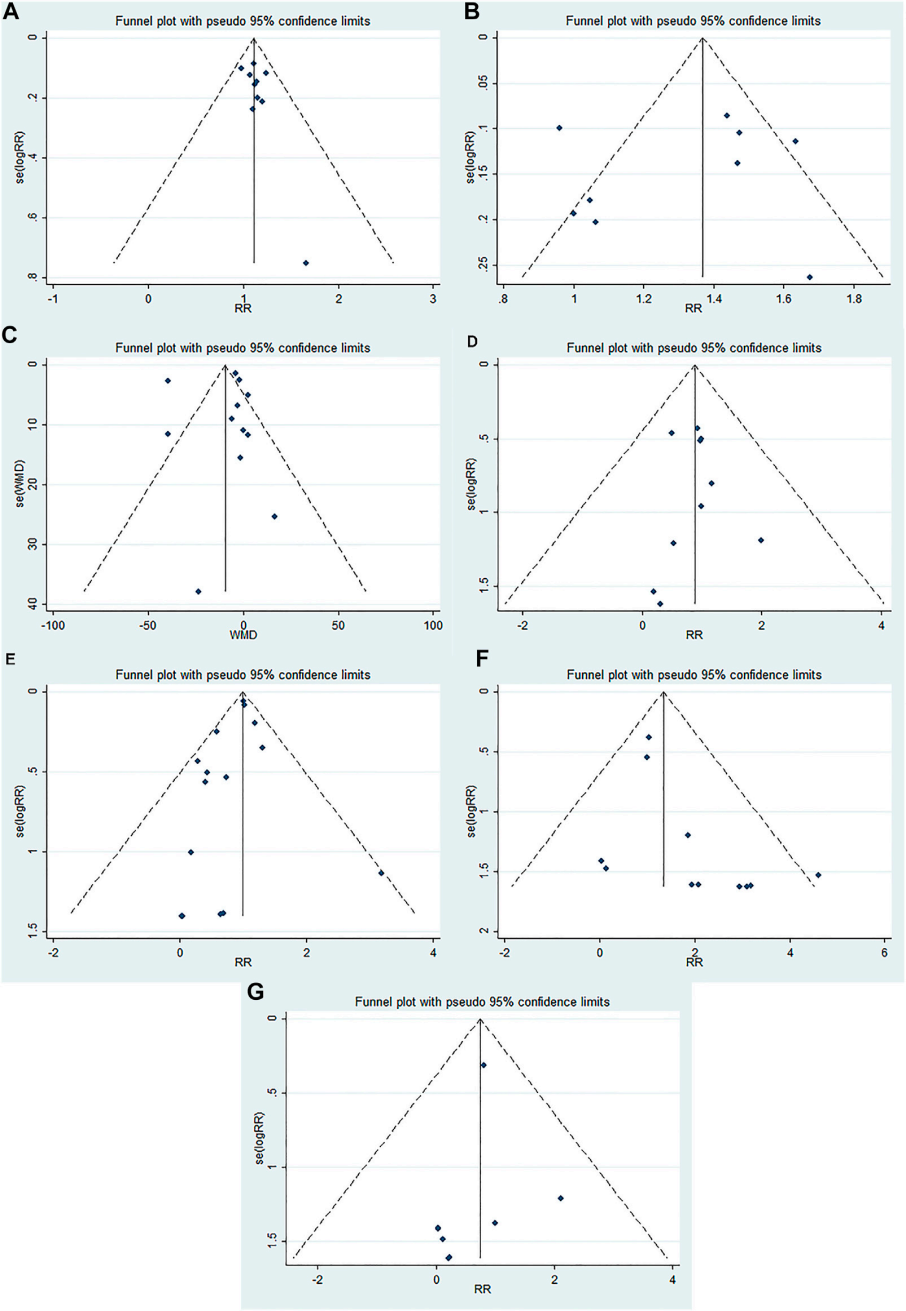
Funnel plots evaluating publication bias for different outcomes: **(A)** OHE improvement, **(B)** prevention of recurrent HE, **(C)** blood ammonia level, **(D)** mortality, **(E)** total adverse events, **(F)** abdominal pain, and **(G)** diarrhea.

The funnel plots were almost symmetric in the outcomes (≥10 RCTs), including OHE improvement, prevention of recurrent HE, mortality, blood ammonia level, total adverse events, abdominal pain, and diarrhea. Furthermore, the Egger’s test showed no statistically significant difference in these outcomes (OHE improvement: *p* = 0.176; prevention of recurrent HE: *p* = 0.463; mortality: *p* = 0.912; blood ammonia level: *p* = 0.360; total adverse effects: *p* = 0.204; abdominal pain: *p* = 0.058; diarrhea: *p* = 0.358). These results indicated no obvious publication bias in our meta-analysis (Figure 3).

### Results of the Meta-analysis

#### Primary Outcomes

##### OHE Improvement

**FIGURE 4:**
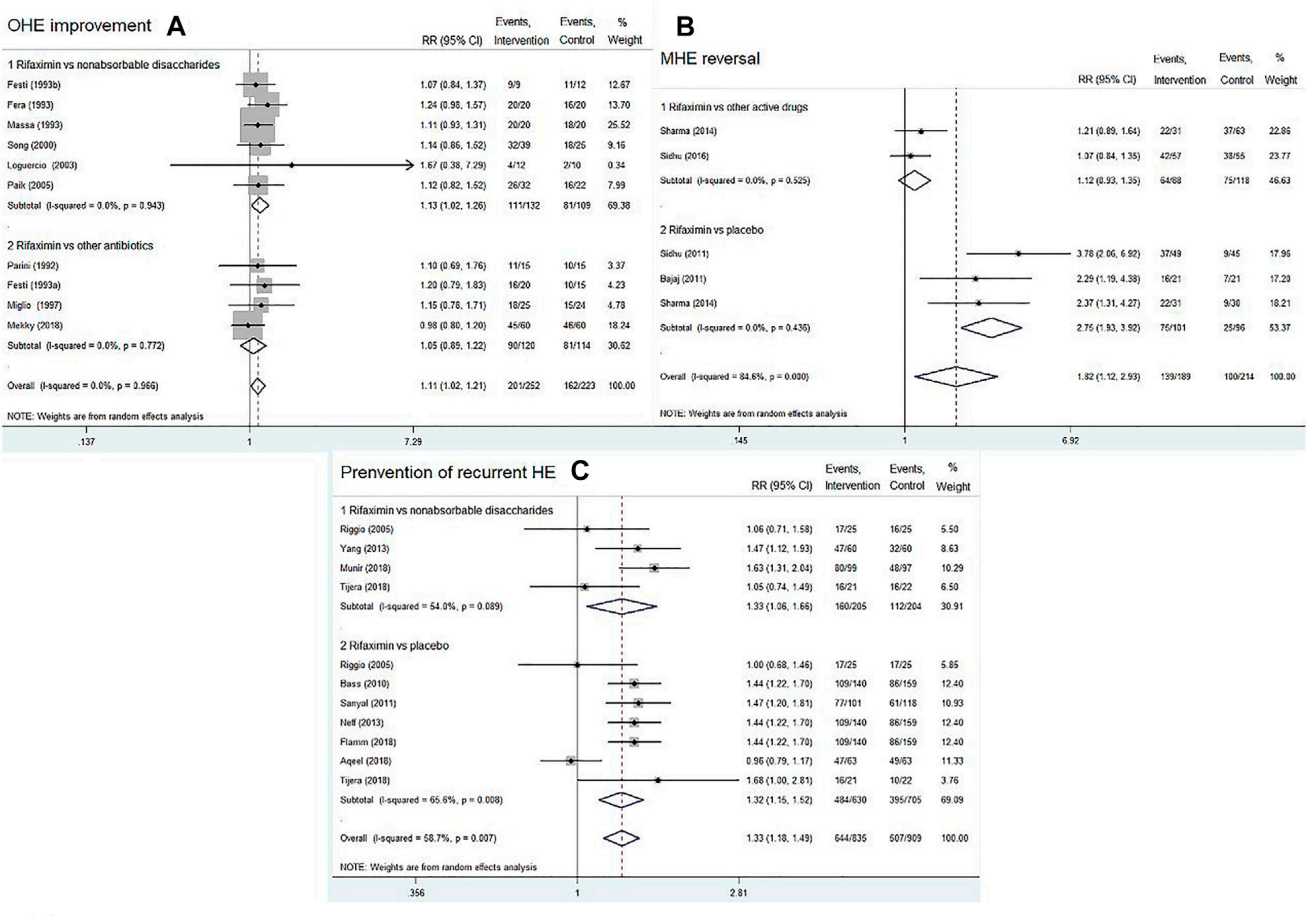
Forest plot of randomized controlled trials on rifaximin vs. placebo or other active drugs on the OHE improvement **(A)**, MHE reversal **(B)**, and prevention of recurrent HE **(C)**.

Ten RCTs reported on OHE improvement of rifaximin compared with that of other active drugs (nonabsorbable disaccharides or other antibiotics). A total of 252 patients were in the rifaximin groups and 223 patients in the control groups. The overall results showed that rifaximin was superior to other active drugs in decreasing HE grade of patients with acute or chronic OHE (RR = 1.11, 95% CI = 1.02–1.21, *p* = 0.022). Further subgroup analysis indicated that rifaximin treatment was better than nonabsorbable disaccharides in terms of OHE improvement (RR = 1.13; 95% CI = 1.02–1.26, *p* = 0.017), whereas there was no difference between rifaximin and other antibiotics (RR = 1.05, 95% CI = 0.89–1.22, *p* = 0.575), Figure 4A. We also found that with the increasing treatment duration, the trend of OHE grade was gradually improved (Supplementary Figure S1A).

##### MHE Reversal

Four RCTs compared rifaximin with placebo or other active drugs in treatment of MHE reversal. After treatment for 1–6 months, rifaximin significantly improved the cognitive impairments in patients with MHE (RR = 1.82, 95% CI = 1.12–2.93, *p* = 0.015). Subgroup analysis indicated that rifaximin treatment was superior to placebo in terms of MHE reversal (RR = 2.75, 95% CI = 1.93–3.92, *p* < 0.01), whereas no significant difference was observed between rifaximin and other active drugs (RR = 1.12, 95% CI = 0.93–1.35, *p* = 0.244), Figure 4B.

##### Prevention of Recurrent HE

Nine RCTs reported the data concerning rifaximin vs*.* the controls (placebo or nonabsorbable disaccharides) for the prevention of recurrent HE. The pooled data revealed that rifaximin significantly reduced the risk of a breakthrough episode compared with the controls (RR = 1.33, 95% CI = 1.18–1.49, *p* < 0.01). Similar findings were observed in subgroup analysis (rifaximin *vs.* nonabsorbable disaccharides: RR = 1.33, 95% CI = 1.06–1.66, *p* = 0.012; rifaximin *vs.* placebo: RR = 1.32, 95% CI = 1.15–1.52, *p* < 0.01), as shown in Figure 4C. We also found that with the increasing treatment duration, the trend of recurrent episodes was gradually decreased (Supplementary Figure S1B).

##### Mortality

**FIGURE 5:**
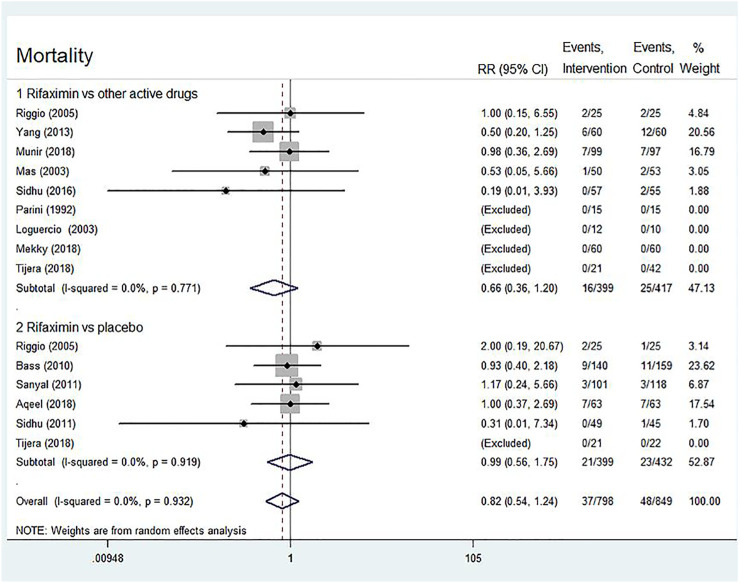
Forest plot of randomized controlled trials on rifaximin treatment for HE. The outcome measure was mortality. The control groups received placebo or other active drugs.

Thirteen trials reported on the mortality risk comparing the rifaximin treatment (*n* = 798) with the controls (placebo or other active drugs, *n* = 849) in patients with different types of HE. The results of both overall and subgroup analyses showed no statistical difference in mortality risk between the two groups (overall: RR = 0.82, 95% CI = 0.54–1.24, *p* = 0.340; rifaximin *vs.* other active drugs: RR = 0.66, 95% CI = 0.36–1.20, *p* = 0.176; rifaximin *vs.* placebo: RR = 0.99, 95% CI = 0.56–1.75, *p* = 0.974, Figure 5).

##### Adverse Events

**FIGURE 6:**
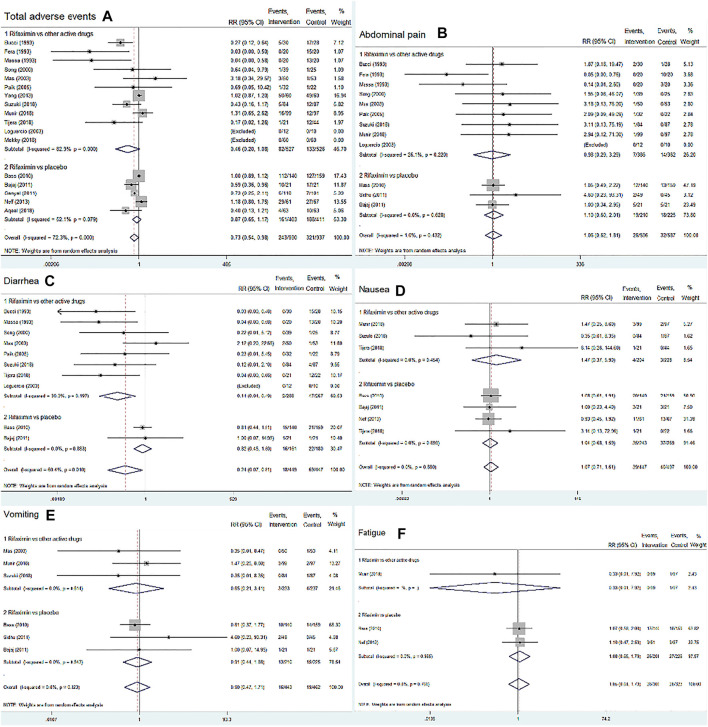
Forest plot of randomized controlled trials on the safety of rifaximin treatment for HE. The outcome measures included total adverse events, abdominal pain, diarrhea, nausea, vomiting and fatigue. The control groups received placebo or other active drugs. **(A)** Total adverse events; **(B)** Abdominal pain; **(C)** Diarrhea; **(D)** Nausea; **(E)** Vomiting; **(F)** Fatigue.

We included 17 RCTs (*n* = 1867) and assessed the incidence of total adverse events and five common adverse events related to drug treatment, including abdominal pain, nausea, fatigue, diarrhea, and vomiting. The overall summary statistics showed no difference in the risk of total adverse events between the rifaximin and control groups (RR = 0.73, 95% CI = 0.54–0.98, *p* = 0.036, Figure 6A). The subgroup analysis also found that rifaximin decreased the incidence of diarrhea compared with other active drugs (RR = 0.14, 95% CI = 0.04–0.49, *n* = 555, *p* = 0.002), whereas no difference was observed in other four adverse events between the two groups (*p* > 0.05), as shown in Figures 6B–F.

#### Secondary Outcomes

##### Blood Ammonia Level

Patients in 12 included RCTs who received rifaximin (*n* = 371) were observed lower blood ammonia levels in comparison to patients who received other active drugs (*n* = 365, nonabsorbable disaccharides and other antibiotics), although the difference did not reach statistical significance (WMD = −8.63, 95% CI = −19.94 to −2.68, *p* = 0.135, Supplementary Figure S2A).

##### Psychometric Indicators

The changes in mental state, flapping tremor and PSE index were observed in both treatment groups, rifaximin vs. other active drugs (nonabsorbable disaccharides and other antibiotics). The overall improvement in psychometric indicators between the two drug groups was statistically significant favouring the use of rifaximin (mental state: WMD = −0.30, 95% CI = −0.53 to −0.06, *n* = 458, *p* = 0.014; flapping tremor: WMD = −0.28, 95% CI = −0.51 to −0.05, *n* = 396, *p* = 0.017; PSE index: WMD = −1.84, 95% CI = −3.37 to −0.30, *n* = 388, *p* = 0.019), as summarized in Supplementary Figure S2B–D.

##### Rehospitalization

Our meta-analysis showed a statistically significant decreased in the frequency of rehospitalization in rifaximin-treated groups compared with placebo or lactulose-treated groups (RR = 0.58, 95% CI = 0.40 to 0.85, *n* = 615, *p* = 0.004, Supplementary Figure S2E).

## Discussion

### Findings and Interpretations

This meta-analysis pooled the data of 28 RCTs that involved a total of 2,979 patients with HE and compared rifaximin treatment vs. placebo or other active drugs. Our results demonstrated that rifaximin had a significant beneficial effect on improvement of OHE, reversal of MHE, and prevention of recurrent HE compared with placebo. Rifaximin treatment was superior to other active drugs in decreasing HE grade, preventing a breakthrough episode, improving psychometric indicators and reducing the frequency of rehospitalisation. Furthermore, rifaximin treatment was found to be safe and well-tolerated by patients with different types of HE. No significance in morality and main adverse effects was observed between rifaximin and the controls. The included trials reported that rifaximin caused abdominal pain in 4.4% (26/596) of the patients (Bucci and Palmieri, 1993; Fera et al., 1993; Massa et al., 1993; Song et al., 2000; Loguercio et al., 2003; Mas et al., 2003; Paik et al., 2005; Bass et al., 2010; Bajaj et al., 2011; Sidhu et al., 2011; Munir et al., 2018; Suzuki et al., 2018), diarrhea in 4.0% (18/449) (Bucci and Palmieri, 1993; Massa et al., 1993; Song et al., 2000; Loguercio et al., 2003; Mas et al., 2003; Paik et al., 2005; Bass et al., 2010; Bajaj et al., 2011; Higuera-de-la-Tijera et al., 2018), nausea in 8.7% (39/447) (Bass et al., 2010; Bajaj et al., 2011; Neff et al., 2013; Higuera-de-la-Tijera et al., 2018; Munir et al., 2018; Suzuki et al., 2018), vomiting in 3.6% (16/443) (Mas et al., 2003; Bass et al., 2010; Bajaj et al., 2011; Sidhu et al., 2011; Munir et al., 2018; Suzuki et al., 2018), fatigue in 8.7% (26/300) (Bass et al., 2010; Neff et al., 2013; Munir et al., 2018), peripheral oedema in 14.9% (30/201) (Bass et al., 2010; Neff et al., 2013), and Clostridium infection in 1.5% (3/200) (Bass et al., 2010; Yang, 2013)during the treatment period.

### Comparison With Other Systematic Reviews

To date, several meta-analyses have assessed the therapeutic effects of rifaximin vs. control interventions on patients with HE (Jiang et al., 2008; Eltawil et al., 2012; Hu and Tang, 2013; Wu et al., 2013; Kimer et al., 2014; Wang, 2015; Zhuo et al., 2019). These systematic reviews included 3–19 trials (*n* = 264–1,370 patients) that were published between 1985 and 2017. In contrast, our meta-analysis had the largest sample size on this topic that included 28 RCTs with 2,979 patients and comprehensively evaluated the efficacy and safety of rifaximin treatment for different types of HE. Moreover, we involved a variety of outcomes in terms of clinical efficacy and safety. Similar to the findings of three previous reviews, we found that rifaximin can significantly reverse MHE and prevent recurrent HE compared with placebo (our results: RR = 2.75, 95% CI: 1.93–3.92, *p* < 0.01; RR = 1.33, 95% CI: 1.18–1.49, *p* < 0.01; Kimer [2014]: RR = 1.32, 95% CI: 1.06–1.65, *p* < 0.01; Hu [2013]: RR = 2.24, 95% CI: 1.20–4.17, *p* = 0.01; Wang [2015]: RR = 0.32, 95% CI: 0.23–0.46, *p* < 0.01) (Hu and Tang, 2013; Kimer et al., 2014; Wang, 2015). Unlike the findings of other three meta-analyses (Wu [2013], Jiang [2008], and Etawil [2012]) (Jiang et al., 2008; Eltawil et al., 2012; Wu et al., 2013), our results showed that rifaximin was superior to other active drugs in improving HE clinical syndrome (HE grade and PSE index). By contrast, they concluded that clinical efficacy of rifaximin was equivalent to that of other oral drugs [Wu (2013): RR = 1.06, 95% CI: 0.94–1.19; *p* = 0.34; Jiang (2008): RR = 1.08, 95% CI: 0.85–1.38, *p* = 0.53; Eltawil (2012): OR = 0.96, 95% CI: 0.94–4.08] (Jiang et al., 2008; Eltawil et al., 2012; Wu et al., 2013). Kimer (2014) reported that rifaximin could significantly reduce the mortality of patients with HE compared with nonabsorbable disaccharides (RR: 0.68, 95% CI: 0.48–0.97, *p* < 0.05) (Kimer et al., 2014). By contrast, we did not find statistically significant difference in mortality between the two groups. Furthermore, we assessed the risk of bias, GRADE evidence, and publication bias for all included studies, which indicated that our results were stable and reliable. By contrast, most of the previous meta-analyses did not conduct these analyses to evaluate the quality of evidence.

### Strengths and Limitations

This meta-analysis has several strengths. First, we updated the existing evidence on HE unlike the previous reviews. Second, we performed a systematic and comprehensive search in all relevant databases without language limitations and rigorously screened, identified, and included appropriate studies. Third, we evaluated a large sample size that included 2,979 patients in 28 RCTs published from 1991 to 2018. Fourth, we conducted appropriate subgroup analyses for primary outcomes, such as effectiveness, mortality, and adverse events, of rifaximin treatment according to type of comparators (rifaximin vs. placebo or other active drugs). Finally, we assessed the quality of evidence for each individual outcome to make our results more reliable.

Nevertheless, this systematic review has few limitations. First, 12 RCTs with unclear risk of bias (i.e., random sequence generation, allocation concealment, incomplete outcome data, and selective reporting) and 12 RCTs with no blinding included herein might have influenced the reliability of the results. Moreover, several included RCTs did not report the methods of sample size calculation, which might have influenced the statistical power of the results for the main effect estimates. However, the results of sensitivity analyses revealed that the effect estimates did not change after excluding these RCTs. Second, we used a random-effect model for this meta-analysis that caused wider confidence intervals and gave more weight to smaller studies. Hence, this model might have potentially expanded the effects of bias in these studies. Lastly, funnel plots and Egger’s test were not performed to assess publication bias of some outcomes with less than 10 RCTs included.

In summary, our meta-analysis updated existing evidence and demonstrated that rifaximin therapy is effective on and well-tolerated by patients with liver cirrhosis and different types of HE. Further subgroup analysis highlighted that the effect of rifaximin was more favourable in improving OHE grade, preventing recurrent HE, improving clinical sign and symptom, and decreasing the incidence of total adverse events than conventional active drugs such as nonabsorbable disaccharides. All the primary and secondary outcomes from the included RCTs belonged to moderate- or high-quality evidence. A recent RCT reported no significant difference between low-dose (440 mg/day) and high-dose (1,100 mg/day) rifaximin treatment (*p* = 0.57) in primary prophylaxis of patients with HE (Sarwar et al., 2019). By contrast, the conventional therapeutic dose of rifaximin was 1,100–1,200 mg/day for HE treatment in the RCTs included herein. Therefore, future trials are warranted to determine the optimal dose of rifaximin for different types of HE.

## Data Availability

The original contributions presented in the study are included in the article/Supplementary Material, further inquiries can be directed to the corresponding authors.

## Abbreviations

HE, hepatic encephalopathy; OHE, overt HE; MHE, minimal HE; RR, risk ratio; CI, confidence interval; NP, neuro-psychometric; CFF, critical flicker frequency; LOLA, L-ornithine-L-aspartate; MD, mean difference; RCT, randomized controlled trial; ROB, risk of bias; PSE, portosystemic encephalopathy; WMD, weighted mean difference.
