# Supplementary material for: Efficacy and Safety of Rifaximin Versus Placebo or Other Active Drugs in Critical ill Patients With Hepatic Encephalopathy
Source: Front Pharmacol. 2021 Oct 8;12:696065. doi: 10.3389/fphar.2021.696065 (PMC8533823; doi:10.3389/fphar.2021.696065)
Supplement: Supplementary file 1 [file Table1.DOCX]

**Supplementary TABLE 1. Detailed Search Strategy**

**Text S1 Search strategy**

**Database: Pubmed from inception to Present> (Search date: January 31, 2021)**

**Search Strategy:**

--------------------------------------------------------------------------------

***Rifaximin terms:***

1 "Rifaximin"[Mesh]

2 (Rifaximin or (4-Deoxy-4'-methylpyrido (1',2'-1,2) imidazo (5,4C) rifamycin) or L 105 or L105 or L-105 or redactiv or xifaxan or normix or rifamycin) [Title/Abstract]

3 or/1-2

***Hepatic Encephalopathy terms:***

4"Hepatic Encephalopathy"[Mesh]

5 (Hepatic Encephalopathy) [Title/Abstract]

6 (Hepatic Encephalopathy or Stupors, Hepatic) [Title/Abstract]

7 (Hepatic Stupor) [Title/Abstract]

8 (Stupor, Hepatic) [Title/Abstract]

9 (Hepatic Stupors) [Title/Abstract]

10 (Hepatic Coma)[Title/Abstract]

11 (Comas, Hepatic)[Title/Abstract]

12 (Coma, Hepatic) [Title/Abstract]

13 (Hepatic Comas) [Title/Abstract]

14 (Encephalopathies, Portosystemic) [Title/Abstract]

15 (Portal-Systemic Encephalopathies) [Title/Abstract]

16 (Portal-Systemic Encephalopathy) [Title/Abstract]

17 (Portal Systemic Encephalopathy) [Title/Abstract]

18 (Encephalopathy, Portal Systemic) [Title/Abstract]

19 (Encephalopathy, Portosystemic) [Title/Abstract]

20 (Encephalopathies, Hepatic) [Title/Abstract]

21 (Encephalopathy, Hepatic) [Title/Abstract]

22 (Hepatocerebral Encephalopathy) [Title/Abstract]

23 (Hepatic Encephalopathies) [Title/Abstract]

24 (Hepatocerebral Encephalopathies) [Title/Abstract]

25 (Encephalopathy, Hepatocerebral) [Title/Abstract]

26 (Portosystemic Encephalopathies) [Title/Abstract]

27 (Encephalopathy, Portal-Systemic) [Title/Abstract]

28 (Encephalopathies, Portal-Systemic) [Title/Abstract]

29 (Encephalopathies, Hepatocerebral) [Title/Abstract]

30 (Portosystemic Encephalopathy) [Title/Abstract]

31 (Fulminant Hepatic Failure with Cerebral Edema) [Title/Abstract]

32 or/4-31

***Final search results: Combining Rifaximin and Hepatic Encephalopathy:***

33 3 and 32 (386)

**Text S2 Search strategy**

**Database: EMBASE (Search date: January 31, 2021)**

**Search Strategy:**

--------------------------------------------------------------------------------

***Rifaximin terms:***

1 'Rifaximin':ab,ti

2 ((4-Deoxy-4'-methylpyrido (1',2'-1,2) imidazo(5,4C) rifamycin) or L 105 or L105 or L-105 or redactiv or xifaxan or normix or rifamycin): ab,ti

3 or/1-2

***Hepatic Encephalopathy terms:***

4 ' Hepatic Encephalopathy ':ab,ti

5 'stupors, hepatic':ab,ti

6 'hepatic stupor':ab,ti

7 'stupor, hepatic':ab,ti

8 'hepatic stupors':ab,ti

9 'hepatic coma':ab,ti

10 'comas, hepatic':ab,ti

11 'coma, hepatic':ab,ti

12 'hepatic comas':ab,ti

13 'encephalopathies, portosystemic':ab,ti

14 'portal-systemic encephalopathies':ab,ti

15 'portal-systemic encephalopathy':ab,ti

16 'portal systemic encephalopathy':ab,ti

17 'encephalopathy, portal systemic':ab,ti

18 'encephalopathy, portosystemic':ab,ti

19 'encephalopathies, hepatic':ab,ti

20 'encephalopathy, hepatic':ab,ti

21 'hepatocerebral encephalopathy':ab,ti

22 'hepatic encephalopathies':ab,ti

23 'hepatocerebral encephalopathies':ab,ti

24 'encephalopathy, hepatocerebral':ab,ti

25 'portosystemic encephalopathies':ab,ti

26 'encephalopathy, portal-systemic':ab,ti

27 'encephalopathies, portal-systemic':ab,ti

28 'encephalopathies, hepatocerebral':ab,ti

29 'portosystemic encephalopathy':ab,ti

30 'fulminant hepatic failure with cerebral edema':ab,ti

31 or/4-30

***Final search results: Combining Rifaximin and Hepatic Encephalopathy:***

7 3 and 31 (839)

**Text S3 Search strategy**

**Database: Web of Science from inception to Present> (Search date: January 31, 2021)**

**Search Strategy:**

--------------------------------------------------------------------------------

***Rifaximin terms:***

1 TOPIC: (Rifaximin)

2 TOPIC: ((4-Deoxy-4'-methylpyrido (1',2'-1,2) imidazo (5,4C) rifamycin) OR L 105 OR L105 OR L-105 OR redactiv OR xifaxan OR normix OR rifamycin)

3 or/1-2

***Hepatic Encephalopathy terms:***

4 TOPIC: (Hepatic Encephalopathy)

5 TOPIC: (Stupors, Hepatic)

6 TOPIC: (Hepatic Stupor)

7 TOPIC: (Stupor, Hepatic)

8 TOPIC: (Hepatic Stupors)

9 TOPIC: (Hepatic Coma)

10 TOPIC: (Comas, Hepatic)

11 TOPIC: (Coma, Hepatic)

12 TOPIC: (Hepatic Comas)

13 TOPIC: (Encephalopathies, Portosystemic)

14 TOPIC: (Portal-Systemic Encephalopathies)

15 TOPIC: (Portal-Systemic Encephalopathy)

16 TOPIC: (Portal Systemic Encephalopathy)

17 TOPIC: (Encephalopathy, Portal Systemic)

18 TOPIC: (Encephalopathy, Portosystemic)

19 TOPIC: (Encephalopathies, Hepatic)

20 TOPIC: (Encephalopathy, Hepatic)

21 TOPIC: (Hepatocerebral Encephalopathy)

22 TOPIC: (Hepatic Encephalopathies)

23 TOPIC: (Hepatocerebral Encephalopathies)

24 TOPIC: (Encephalopathy, Hepatocerebral)

25 TOPIC: (Portosystemic Encephalopathies)

26 TOPIC: (Encephalopathy, Portal-Systemic)

27 TOPIC: (Encephalopathies, Portal-Systemic)

28 TOPIC: (Encephalopathies, Hepatocerebral)

29 TOPIC: (Portosystemic Encephalopathy)

30 TOPIC: (Fulminant Hepatic Failure with Cerebral Edema)

31 or/4-30

***Final search results: Combining Rifaximin and Hepatic Encephalopathy:***

31 3 and 31 (827)

**Text S4 Search strategy**

**Database: Cochrane Library from inception to Present> (Search date: January 31, 2021)**

**Search Strategy:**

--------------------------------------------------------------------------------

***Rifaximin terms:***

1 MeSH descriptor: [Rifaximin] explode all trees

2 ((4-Deoxy-4'-methylpyrido (1',2'-1,2) imidazo (5,4C) rifamycin) or L 105 or L105 or L-105 or redactivor xifaxan or normix or rifamycin): ti,ab,kw (Word variations have been searched)

3 or/1-2

***Hepatic Encephalopathy terms:***

4 MeSH descriptor: [Hepatic Encephalopathy] explode all trees

5 (Hepatic Encephalopathy or Stupors, Hepatic):ti,ab,kw (Word variations have been searched)

6 (Hepatic Stupor):ti,ab,kw (Word variations have been searched)

7 (Stupor, Hepatic):ti,ab,kw (Word variations have been searched)

8 (Hepatic Stupors):ti,ab,kw (Word variations have been searched)

9 (Hepatic Coma):ti,ab,kw (Word variations have been searched)

10 (Comas, Hepatic):ti,ab,kw (Word variations have been searched)

11 (Coma, Hepatic) :ti,ab,kw (Word variations have been searched)

12 (Hepatic Comas):ti,ab,kw (Word variations have been searched)

13 (Encephalopathies, Portosystemic) :ti,ab,kw (Word variations have been searched)

14 (Portal-Systemic Encephalopathies):ti,ab,kw (Word variations have been searched)

15 (Portal-Systemic Encephalopathy):ti,ab,kw (Word variations have been searched)

16 (Portal Systemic Encephalopathy):ti,ab,kw (Word variations have been searched)

17 (Encephalopathy, Portal Systemic):ti,ab,kw (Word variations have been searched)

18 (Encephalopathy, Portosystemic):ti,ab,kw (Word variations have been searched)

19 (Encephalopathies, Hepatic):ti,ab,kw (Word variations have been searched)

20 (Encephalopathy, Hepatic):ti,ab,kw (Word variations have been searched)

21 (Hepatocerebral Encephalopathy):ti,ab,kw (Word variations have been searched)

22 (Hepatic Encephalopathies):ti,ab,kw (Word variations have been searched)

23 (Hepatocerebral Encephalopathies):ti,ab,kw (Word variations have been searched)

24 (Encephalopathy, Hepatocerebral):ti,ab,kw (Word variations have been searched)

25 (Portosystemic Encephalopathies):ti,ab,kw (Word variations have been searched)

26 (Encephalopathy, Portal-Systemic):ti,ab,kw (Word variations have been searched)

27 (Encephalopathies, Portal-Systemic):ti,ab,kw (Word variations have been searched)

28 (Encephalopathies, Hepatocerebral):ti,ab,kw (Word variations have been searched)

29 (Portosystemic Encephalopathy):ti,ab,kw (Word variations have been searched)

30 (Fulminant Hepatic Failure with Cerebral Edema):ti,ab,kw (Word variations have been searched)

31 or/4-30

***Final search results: Combining Rifaximin and Hepatic Encephalopathy:***

31 3 and 31 (261)

**Text S5 Search strategy**

**Chinese databases: CNKI,VIP, and Wanfang from inception to Present> (Search date: January 31, 2021)**

**Search Strategy:**

--------------------------------------------------------------------------------

***Rifaximin terms:***

1 SU = '利福昔明'

***Hepatic Encephalopathy terms:***

2 SU = '肝性脑病'

3 SU = '脑病'

4 SU = '肝性'

5 SU = '门体脑病'

6 or/2-5

***Final search results: Combining Rifaximin and Hepatic Encephalopathy:***

7 1 and 6 (127)
