# Supplementary figures and images for: Efficacy and Safety of Rifaximin Versus Placebo or Other Active Drugs in Critical ill Patients With Hepatic Encephalopathy
Source: Front Pharmacol. 2021 Oct 8;12:696065. doi: 10.3389/fphar.2021.696065 (PMC8533823; doi:10.3389/fphar.2021.696065)

Blood ammonia level (A)

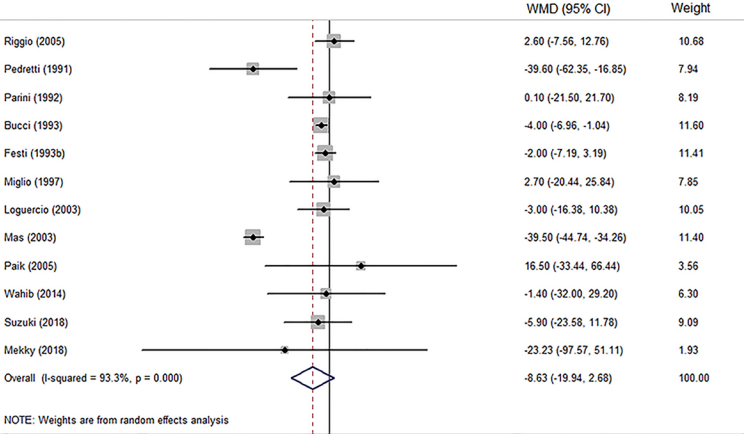

Mental state (B)

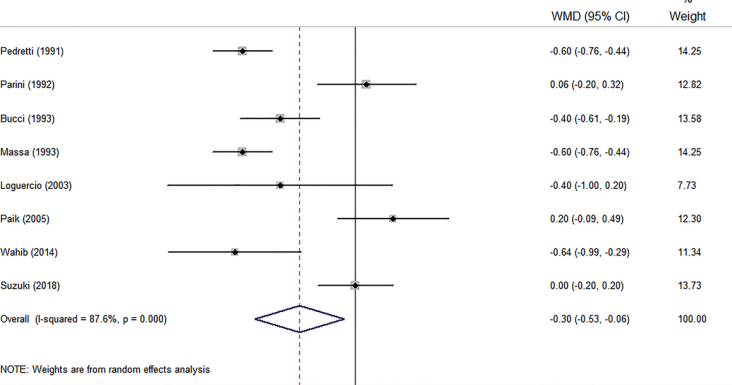

Flapping tremor (C)

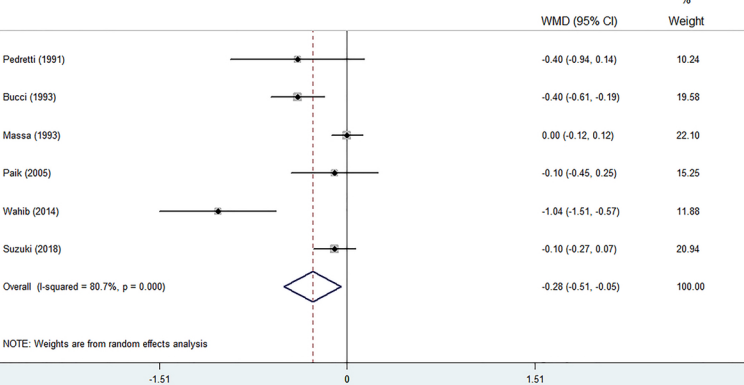

PSE index (D)

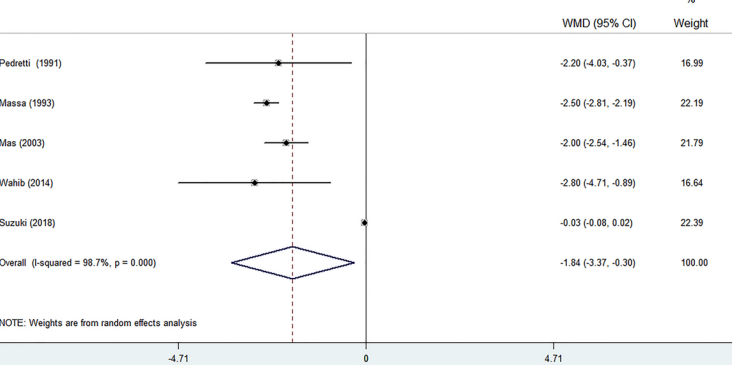

Rehospitalization (E)

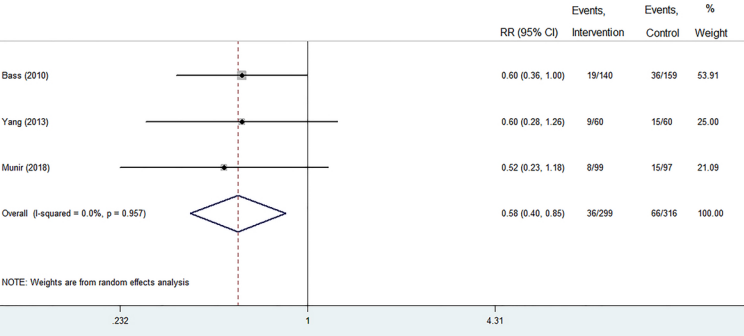

Supplement: Supplementary file 2 [file Image2.PDF]

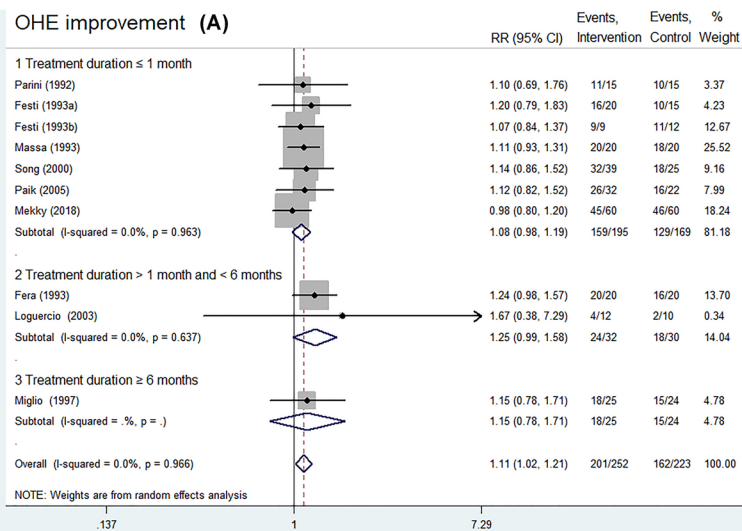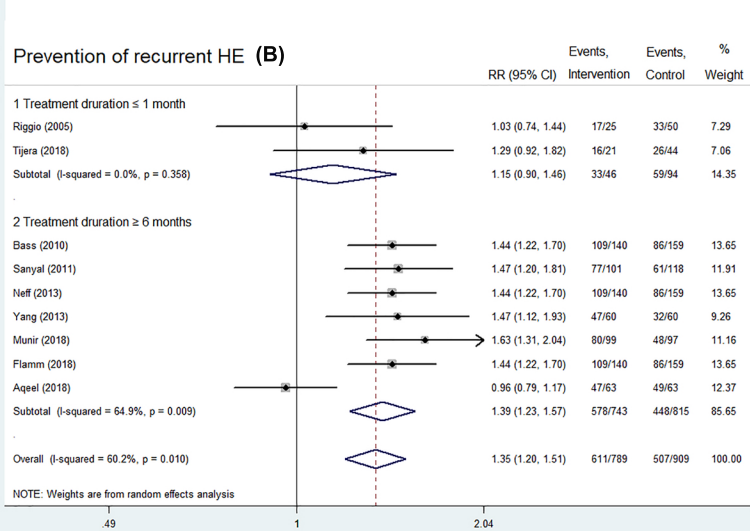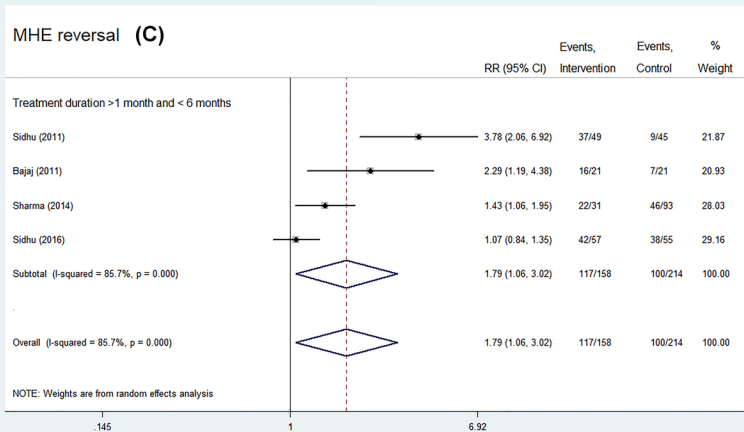

Supplement: Supplementary file 6 [file Image1.PDF]
