# Supplementary material for: Efficacy and Safety of Rifaximin Versus Placebo or Other Active Drugs in Critical ill Patients With Hepatic Encephalopathy
Source: Front Pharmacol. 2021 Oct 8;12:696065. doi: 10.3389/fphar.2021.696065 (PMC8533823; doi:10.3389/fphar.2021.696065)
Supplement: Supplementary file 3 [file Table2.DOCX]

**Supplementary TABLE 2. Characteristics of included randomized controlled trials.**

| **First author year** | **Study design** | **Participants** | **Type of HE** | **Study groups** | **Treatmentduration** | **Number of**  **patients** | **Outcomes** |
| --- | --- | --- | --- | --- | --- | --- | --- |
| Pedretti  1991^50^ | A double-blind,  randomized,  controlled trial | Patients with  hyperammoniemic state of portal systemic  encephalopathy | OHE (I-III grade) | rifaximin (1200mg/day) *vs* neomycin (3000  mg/day) | 21 days | Intervention:  15 patients  Control:  15 patients | NH_3_level, mental status, asterixis,  EEG, PSE level, *etc*. |
| Parini  1992^51^ | A randomized,  controlled trial | Severe patients with  portal-systemic  encephalopathy | OHE (I-III grade) | rifaximin (1200  mg/day) *vs*  paromomycin (1500 mg/day) | 10 days | Intervention:  15 patients  Control:  15 patients | remission of the clinical signs and  symptoms of HE, ammonia level,  mental state, mortality |
| Massa  1993^37^ | A double-blind,  randomized,  controlled trial | Cirrhotics patients with  HE | OHE (II or III grade) | rifaximin  (1200 mg/day) *vs*  lactulose (60 ml/day) | 15 days | Intervention:  20 patients  Control:  20 patients | HE severity, mental status, EEG,  asterixis, adverse events, *etc*. |
| Fera  1993^38^ | A randomized,  double-blind,  controlled trial | Cirrhotics patients with  HE | OHE (I grade) | rifaximin (1200  mg/day) *vs* lactulose  (120 ml/day) | 3 months | Intervention:  20 patients  Control:  20 patients | PSE severity, EEG, adverse events,  *etc*. |
| Bucci  1993^15^ | A double-blind,  double-dummy,  randomized,  controlled trial | Critical patients with  medium to severe degree  Of porto-systemic  encephalopathy. | OHE (I-III grade) | rifaximin (1200  mg/day) *vs* lactulose  (30 g/day) | 15 days | Intervention:  30 patients  Control:  28 patients | mental state, asterixis, EEG, serum  ammonia concentrations, adverse  events, *etc.* |
| Festi  1993a^39^ | A multicentre,  randomized,  controlled trial | Cirrhotic patients with clinical and biochemical  signs of HE | OHE (I grade) | rifaximin (1200  mg/day) *vs* neomycin  (3000 mg/day) | 21 days | Intervention:  20 patients  Control:  15 patients | neurologic signs, frequency of  asterixis, EEG, *etc.* |
| Festi  1993b^39^ | A multicentre,  randomized,  controlled trial | Cirrhotic patients with clinical and biochemical  signs of HE | OHE (I grade) | rifaximin (1200  mg/day) *vs* lactulose  (40 g/day) | 21 days | Intervention:  9 patients  Control:  12 patients | neurologic signs, EEG, blood  ammonia level, *etc*. |
| Miglio  1997^52^ | A double-blind,  randomized,  controlled,  multicentre trial | Patients with a definite diagnosis of cirrhosis | OHE (I or II grade) | rifaximin (1200  mg/day) *vs* neomycin  (3 g/day) | 6 months | Intervention:  25 patients  Control:  24 patients | HE grade, blood ammonial evel,  Reitan test, *etc*. |
| Song  2000^40^ | A one-blind,  randomized,  controlled trial | Cirrhotics patients with  HE | OHE (I-III grade) | rifaximin (1200  mg/day) *vs* lactulose  (90 ml/day) | 7 days | Intervention:  39 patients  Control:  25 patients | clinical efficacy, adverse events, HE  index, *etc*. |
| Mas  2003^14^ | A double-blind,  double-dummy,  randomized,  controlled trial | Patients with grade I–III acute hepatic  encephalopathy | OHE (I-III grade) | rifaximin (1200  mg/day) *vs* lactitol  (60 g/day) | 5-10 days | Intervention:  50 patients  Control:  53 patients | clinical efficacy, ammoniemia, EEG, PSE index, mortality, adverse events |
| Loguercio  2003^41^ | A double-blind,  randomized,  controlled trial |  | OHE (II-III grade) | rifaximin (1200  mg/day) *vs* lactitol  (60 g/day) | 3 months | Intervention:  12 patients  Control:  10 patients | HE degree, blood ammonia level,  mental state, mortality, adverse  events |
| Riggio  2005^45^ | A randomized,  controlled trial | Patients with  Liver cirrhosis and HE  Submitted to TIPS | Recurrent HE | rifaximin (1200  mg/day) *vs* lactitol  (60ml/day) or placebo | 30 days | Intervention:  25 patients  Control:  50 patients | episode of overt HE, TMT-A,  mortality, *etc*. |
| Paik  2005^30^ | An open-label,  prospective,  randomized  trial | Patients with  liver cirrhosis and HE | OHE (I-III grade) | rifaximin (1200  mg/day) *vs* lactulose  (90 ml/day) | 7 days | Intervention:  32 patients  Control:  22 patients | HE grade, blood NH_3_ level, mental  status grade, grade of flapping  tremor, adverse effects, *etc*. |
| Bass  2010^28^ | A double-blind,  randomized,  controlled trial | Patients who were in remission from recurrent  HE resulting from chronic liver disease | Recurrent HE | rifaximin (1100  mg/day) *vs* placebo | 6 months | Intervention:  140 patients  Control:  159 patients | breakthrough episodes of HE, safety,  hospitalizations, mortality |
| Sidhu  2011^36^ | A double-blind,  randomized,  controlled trial | Patients with liver  cirrhosis | MHE | rifaximin (1200  mg/day) *vs* placebo | 8 weeks | Intervention:  49 patients  Control:  45 patients | driving errors, development of overt HE, mortality, side effects, *etc*. |
| Sanyal  2011^33^ | A double-blind,  randomized,  controlled trial | Patients with cirrhosis in remission from HE  and a documented history of recurrent HE episodes | Recurrent HE | rifaximin (1100  mg/day) *vs* placebo | 6 months | Intervention:  101 patients  Control:  118 patients | breakthrough HE, MELD score, death, time to hospitalization |
| Bajaj  2011^13^ | A randomized,  double-blind,  controlled trial | Patients with cirrhosis and HE | MHE | rifaximin (1100  mg/day) *vs* placebo | 8 weeks | Intervention:  21 patients  Control:  21 patients | driving performance, psychometric  test performance, quality of life,  adverse events |
| Neff  2013^46^ | A randomized,  double-blind,  controlled trial | Cirrhotic patients with  recurrent HE. | Recurrent HE | rifaximin (1100  mg/day) *vs* placebo | 6 months | Intervention:140 patients  Control:  159 patients | breakthrough overt HE, adverse  events |
| Yang  2013^32^ | A randomized,  controlled trial | Cirrhotic patients with  recurrent HE | Recurrent HE | rifaximin (1100  mg/day) *vs* lactulose (0.667g/ml, 15 ml  twice a day) | 12 months | Intervention:  60 patients  Control：  60 patients | breakthrough episodes of HE,  mortality, hospitalization |
| Sharma  2014^49^ | A randomized,  controlled trial | Cirrhotics patients with  HE | MHE | rifaximin (1200  mg/day) *vs* LOLA (2  sachets 3g each thrice a day), probiotics (one  capsule twice a day) or placebo | 2 months | Intervention: 31 patients  Control:  93 patients | critical flicker frequency values,  number connection test,  neuropsychometric test, *etc*. |
| Wahib  2014^42^ | A randomized,  controlled trial | Patients with I–III grade  HE | OHE (I–III grade) | rifaximin (1200  mg/day) *vs* lactulose  (90 ml daily) | 7 days | Intervention: 25 patients  Control:  25 patients | blood ammonia level, mental state,  grade of flapping tremor, HE index |
| Sidhu  2016^44^ | An open label，randomized,  controlled,  non-inferiority  trial | Cirrhotics patients with  HE | MHE | rifaximin (1200  mg/day) *vs* lactulose  (30-120 ml/day) | 3 months | Intervention: 57 patients  Control:  55 patients | minimal HE reversal, quality of life,  mortality |
| Flamm  2018^47^ | A multicentre,  randomized,  phase III,  controlled trial | Cirrhotics patients with  HE | Recurrent HE | rifaximin (1100  mg/day) *vs*  placebo | 6 months | Intervention: 140 patients  Control:  159 patients | HE episodes, time to a breakthrough episode of overt HE |
| Suzuki  2018^43^ | A multicentre,  randomized, one blind, controlled trial | Patients with grade I or II HE and hyperammonemia | OHE (I or II grade) | rifaximin (1200  mg/day) *vs* lactitol  (18-36 g/day) | 14 days | Intervention: 84 patients  Control:  87 patients | blood ammonia concentration, PSE  index, asterixis grade, mental state,  quality of life, adverse drug  reactions |
| Mekky  2018^53^ | An open labelled  randomized,  controlled trial | Patients with an acute  episode of HE on top of cirrhosis | OHE (I-IV grade) | rifaximin (1200  mg/day) *vs*  metronidazole  (750 mg/day) | 3 days | Intervention:  60 patients  Control:  60 patients | clinical improvement, death, serum  ammonia level, *etc*. |
| Munir  2018^35^ | A randomized,  controlled trial | Cirrhotics patients with  HE | Recurrent HE | rifaximin (1100  mg/day) *vs* lactulose  (30-120 ml/day) | 6 months | Intervention:  99 patients  Control:  97 patients | HE episode, admitted to the hospital with HE, mortality, MELD score,  adverse events, *etc*. |
| Aqeel  2018^48^ | A triple-blind,  randomized,  controlled trial | Patients with chronic liver disease and HE | Recurrent HE | rifaximin (1100  mg/day) *vs* placebo | 6 months | Intervention:  63 patients  Control:  63 patients | patients remained free from HE,  MELD score, death |
| Tijera  2018^34^ | A randomized,  double-blind,  controlled trial | Cirrhotic patients with  variceal bleeding | Recurrent HE | rifaximin (1200  mg/day) *vs* placebo,  lactulose or LOLA | 7 days | Intervention:  21 patients  Control:  66 patients | development of overt HE, mortality, adverse events |

**Abbreviations:** OHE, overt hepatic encephalopathy; MHE, minimal hepatic encephalopathy; PSE, portal-systemic encephalopathy; EEG, electroencephalogram; HE, hepatic encephalopathy; MELD, model for end-stage liver disease; LOLA, L-ornithine-L-aspartate.
