# Supplementary material for: Efficacy and Safety of Rifaximin Versus Placebo or Other Active Drugs in Critical ill Patients With Hepatic Encephalopathy
Source: Front Pharmacol. 2021 Oct 8;12:696065. doi: 10.3389/fphar.2021.696065 (PMC8533823; doi:10.3389/fphar.2021.696065)
Supplement: Supplementary file 4 [file Table3.DOCX]

**Supplementary TABLE 3. Summary of GRADE evidence profile of rifaximin *versus* other interventions.**

**
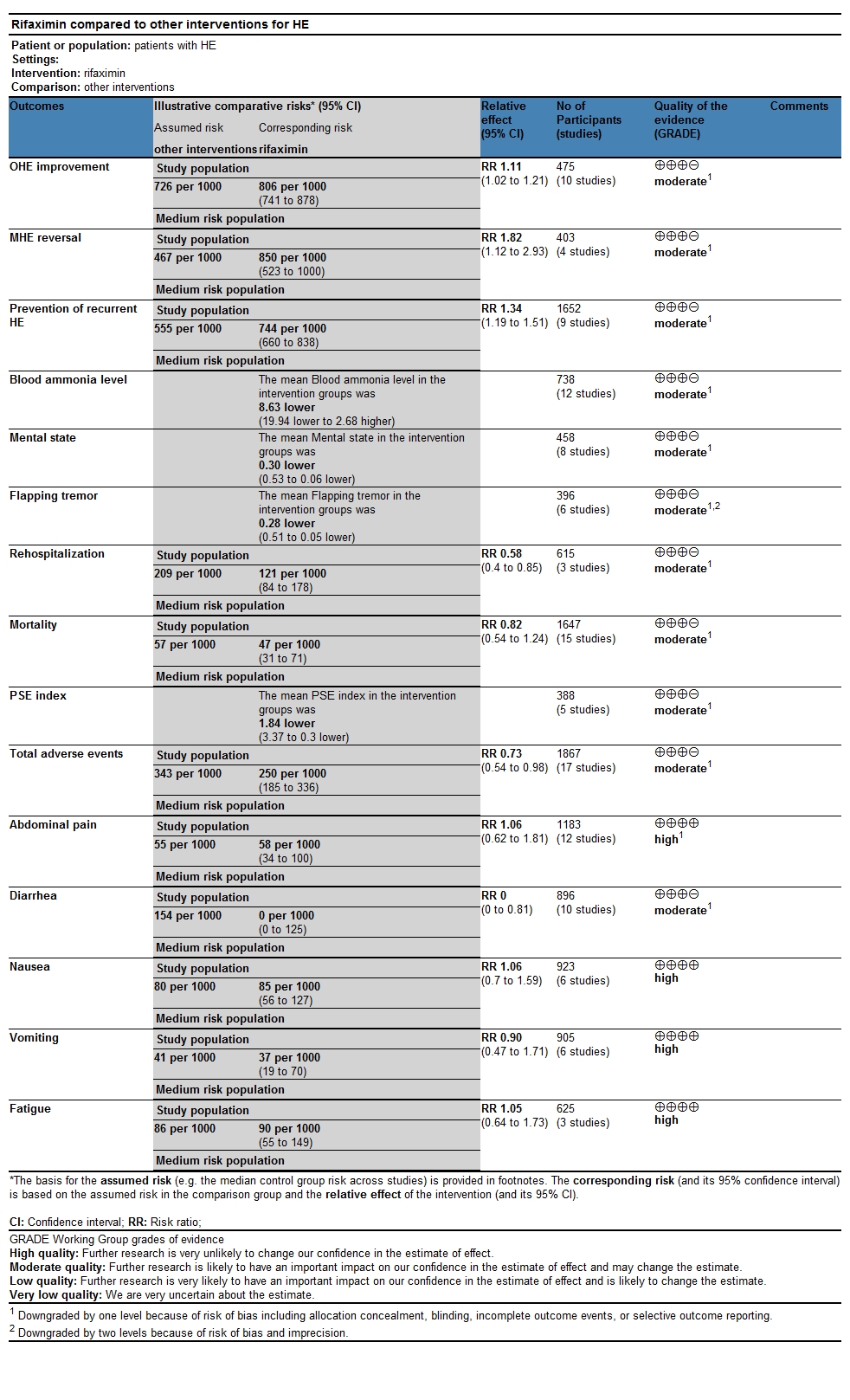
**
