# Supplementary material for: Efficacy and Safety of Rifaximin Versus Placebo or Other Active Drugs in Critical ill Patients With Hepatic Encephalopathy
Source: Front Pharmacol. 2021 Oct 8;12:696065. doi: 10.3389/fphar.2021.696065 (PMC8533823; doi:10.3389/fphar.2021.696065)
Supplement: Supplementary file 5 [file DataSheet1.docx]

**SUPPLEMENTARY FIGURE LEGENDS**

**Supplementary FIGURE 1.** Forest plot of the influence of treatment duration of rifaximin on the primary outcomes of clinical efficacy. The outcome measures included OHE improvement (A), prevention of recurrent HE (B), and MHE reversal (C). The control groups received placebo or other active drugs.

**Supplementary FIGURE 2.** Forest plot of randomized controlled trials on the secondary outcomes of rifaximin treatment for HE. The outcome measures included blood ammonia level (A), mental state (B), flapping tremor (C), PSE index (D), and rehospitalisation (E). The control groups received placebo or other active drugs.
